# Exploring the relationship between physical activity and smartphone addiction among college students in Western China

**DOI:** 10.3389/fpubh.2025.1530947

**Published:** 2025-02-21

**Authors:** Chun Lai, Peiling Cai, Junyi Liao, Xiwei Li, Yuanyuan Wang, Mengping Wang, Peng Ye, Xinwei Chen, Brett D. Hambly, Xiaoping Yu, Shisan Bao, Haifeng Zhang

**Affiliations:** ^1^College of Physical Education, Chengdu University, Chengdu, China; ^2^School of Preclinical Medicine, Chengdu University, Chengdu, China; ^3^Clinical Medical College & Affiliated Hospital, Chengdu University, Chengdu, China; ^4^School of Music and Dance, College of Chinese & ASEAN Art, Chengdu University, Chengdu, China; ^5^Centre for Healthy Futures, Torrens University Australia, Sydney, NSW, Australia; ^6^The Cardiovascular Centre, Foreign Affair Office, The First People's Hospital of Baiyin, Gansu, China; ^7^Third Affiliated Hospital of Gansu University of Chinese Medicine, Gansu, China

**Keywords:** physical activity, exercise intensity, smartphone addiction, college students, Western China, cross-sectional survey

## Abstract

**Background:**

Smartphone addiction (SA) refers to a behavioral disorder characterized by an irresistible compulsion to excessively engage with mobile devices. Currently, the evidence regarding the relationship between physical activity (PA), exercise intensity (EI), and SA is limited, particularly within Chinese populations. This study aims to explore the correlation between PA, EI, and SA, specifically investigating how PA and EI impact SA to better understand the nature of this relationship.

**Methods:**

A cross-sectional study was conducted involving college students from over 20 universities in Western China. Data were collected on participants’ engagement in PA, EI, and SA. Additionally, covariates such as age, gender, ethnicity, academic classification, university location, discipline, year of study, hometown region, sibling status, relationship status, and social interactions were recorded. Multivariate logistic regression models were used to assess the association between PA, EI, and SA. Stratified and interaction analyses were performed to examine whether the relationship remained consistent across different subgroups.

**Results:**

Of the 3,506 college students surveyed, 1,905 (54.3%) experienced SA. The prevalence of SA was 11.3% lower in the group that engaged in PA compared to those who did not. In the fully adjusted model, PA was negatively associated with SA (OR = 0.70, 95% CI: 0.59–0.82, *p* < 0.001). EI was also inversely associated with SA. Moderate- and vigorous-intensity exercise had odds ratios of 0.81 (95% CI: 0.67–0.98, *p* = 0.034) and 0.83 (95% CI: 0.68–1.00, *p* = 0.046), respectively, compared with low-intensity exercise. Similar patterns were observed in subgroup analyses (all *p* values for interaction >0.05).

**Conclusion:**

The findings indicate a significant negative association between PA, EI, and SA, highlighting the potential of promoting PA and higher EI as strategies to reduce SA among college students.

## Introduction

China launched its 5G network services in June 2019, ushering in an era of enhanced data transmission, reduced latency, and expanded connectivity. These innovations have provided significant technological support and opened new opportunities in sectors such as the Internet of Things (IoT), industrial automation, autonomous vehicles, telemedicine, and virtual reality (1). The rapid evolution of mobile networking, along with decreasing smartphone prices, has made smartphones the primary means of internet access. Consequently, smartphone penetration in China has steadily increased, reflecting their growing adoption across the population.

According to the 53rd Statistical Report by the China Internet Network Information Center (CNNIC), China’s internet user base reached 1.092 billion, with an internet penetration rate of 77.5%. Among these, 99.9% were mobile internet users, totaling 1.091 billion. The 10–29 age group constitutes 28.4% of the online population (2), with college students making up 21.0% of users (3). On average, these students spend 26.1 h per week online (2). As internet usage becomes an indispensable part of modern life, the growing reliance on smartphones has led to smartphone addiction (SA), a nonsubstance-related addiction (4, 5) negatively affecting physical and psychological health, social interactions (6), and academic performance, with severe cases linked to suicidal ideation (7).

Physical activity (PA) plays a key role in promoting health and well-being. Research shows PA alleviates stress (8), reduces symptoms of depression and anxiety (9–11), and enhances overall mental health. In college students, PA is an essential countermeasure to sedentary behaviors driven by academic demands and extensive digital engagement (12). It fosters resilience and helps mitigate the adverse effects of prolonged screen time, providing a healthy outlet for stress and maintaining psychological balance.

A recent study showed that college students often fail to meet recommended PA levels, partly due to prioritizing academic success under parental expectations. This highlights the need to promote PA as a protective factor against excessive smartphone use (13).

The psychological effects of SA are well-documented, with research exploring its impact on anxiety (14, 15), depression, stress (15), self-esteem (16), and social isolation (5). However, the relationship between PA and SA remains under-explored. Previous research by our team found an inverse association between PA, exercise intensity (EI), and internet addiction (IA) among college students. Higher PA levels were linked to a lower risk of IA (17), suggesting a similar relationship may exist between PA and SA. This aligns with recent findings showing that PA and sedentary behavior are correlated with academic performance among college students, particularly freshmen (18).

The potential of PA to reduce SA among college students warrants further investigation, especially given the limited evidence in this area.

Western China, as defined by China’s economic and geographical classifications, consists of 12 administrative regions, including Chongqing, Sichuan, Yunnan, and others. These areas account for 27.2% of the country’s population (19), but their economies are less developed compared to Eastern and Southern China.

Western China presents a unique context for studying the relationship between PA and SA. The region is characterized by diverse cultural practices, socio-economic disparities, and distinct lifestyle behaviors. The geographical and economic challenges may influence both PA levels and smartphone usage patterns. Despite these features, Western China has received limited attention in SA and PA research. By focusing on college students in this region, this study aims to fill this gap and provide insights for culturally and regionally tailored interventions to promote healthier behaviors.

A 2015 report highlighted that international Chinese students are particularly vulnerable to SA, which significantly affects them emotionally and financially (20). This vulnerability may stem from the transition to university life and the unfamiliarity of the new environment. A more recent report from Sichuan Province found that SA is closely associated with self-control issues and rumination, especially in the context of psychological distress, such as loneliness (21).

This study aims to explore the correlation between PA, EI, and SA, specifically investigating how PA and EI impact SA to better understand the nature of this relationship.

## Methods

### Sample size calculation of participants

In this study, we determined the necessary sample size using PASS 2021 software for a cross-sectional survey. The corresponding formula for calculating the estimated sample size n is as follows:

$n={Z_{\alpha }}^{2}\times P\times \left(1-P\right)/\delta^{2}$.

Where Z represents the number of standard errors away from the mean, P is the estimated prevalence of the disease, and δ is the desired precision level. The value of P was estimated based on the findings of a recent study conducted in China, which reported an overall prevalence of smartphone addiction (SA) at 54.5% (22). The absolute precision was set at 3.0% for this study, and the value of Z_α_ was 1.96. In total, we aimed to enroll 1,057 participants. Additionally, we planned to include at least 1,113 college students from Western China, in our survey to accommodate a 5% margin of error.

### Sampling strategy

For the current study, we adopted a combination of convenience and snowball sampling methods to conduct a cross-sectional survey. The Quick Response (QR) code of the online survey questionnaire was first distributed to lecturers teaching at various universities in Western China using convenience sampling. These lecturers then shared the QR code with their students, inviting them to voluntarily complete the questionnaire. Subsequently, through snowball sampling, students were encouraged to distribute the QR code to other college students they knew (friends or classmates) who were also enrolled in universities in Western China.

To prevent duplicate responses, the online platform was configured to allow submissions only through WeChat and to limit participation to one response per user. To maintain confidentiality, only the unique online WeChat identifying number, not including the name, was used. Additionally, to avoid incomplete submissions, the platform required respondents to answer all questions before successfully submitting the questionnaire. By combining these two sampling methods, we effectively expanded both the sample size and the sampling range.

### Data collection

This research was conducted from April to May 2024. We disseminated an online survey specifically designed for undergraduate participants using the Questionnaire Star platform.^1^ Participants completed self-reported questionnaires to assess their participation in PA, EI and SA.

Students were drawn from more than 20 universities across Western China (see Supplementary Table 1), which represented a diverse geographical and academic spectrum of Western China.

Before the investigation, we established inclusion and exclusion criteria. The inclusion criteria were: Chinese nationality, being a full-time college undergraduate student, and being able to engage in physical activity. The exclusion criteria were: not Chinese nationality, not being a full-time college student, being a graduate student, or being unable to participate in physical exercise due to physical limitations.

Additionally, we defined the criteria for a valid questionnaire as follows: all questions must be answered, there should be no obvious mistakes (e.g., an age of 80 years), each IP address can be used only once, and the time taken to complete the questionnaire must be between 1.5 and 15 min.

**Figure 1:**
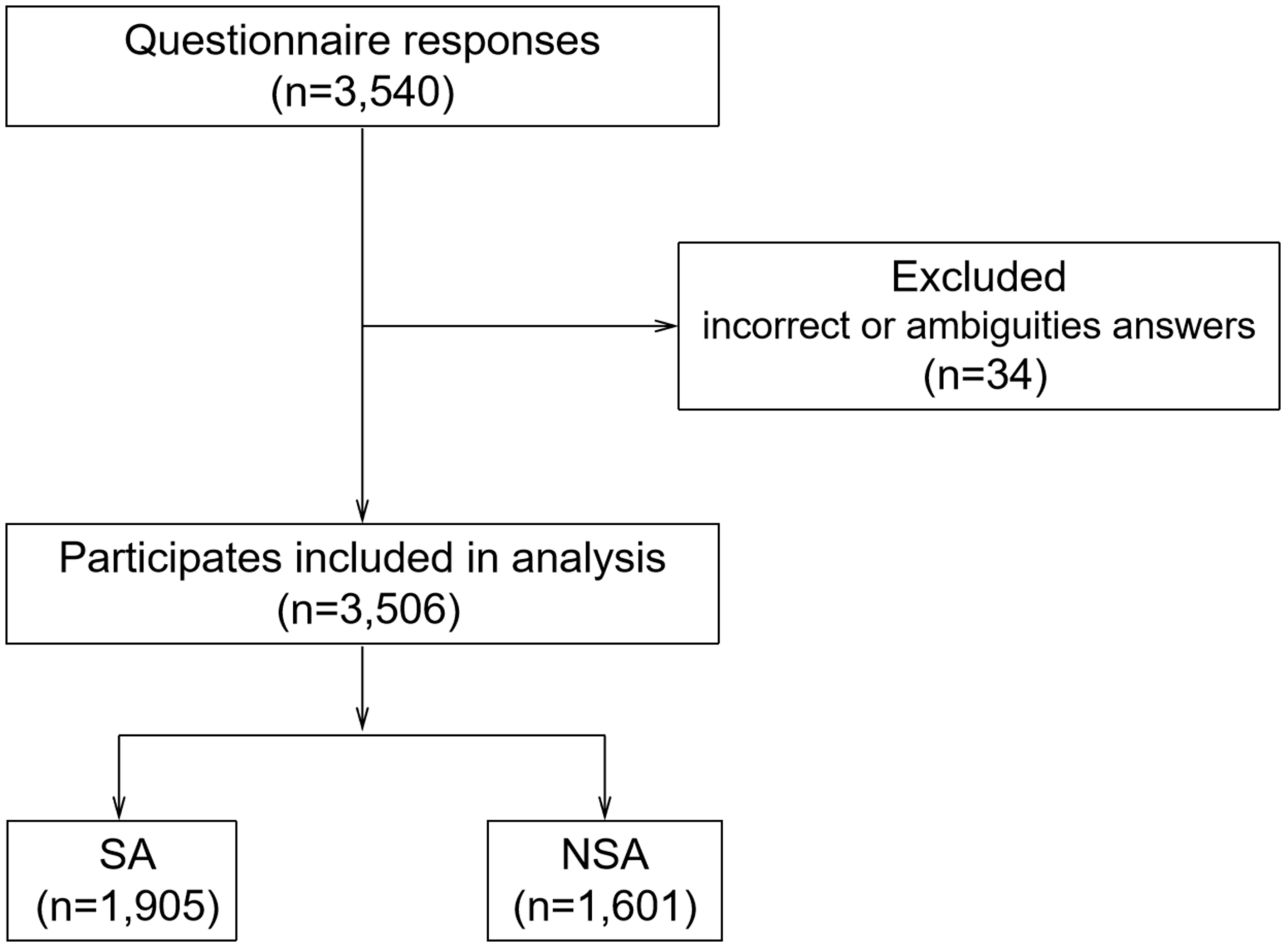
Flowchart of participants selection.

In total, we received 3,540 responses from participants, of which 34 were excluded from our analysis due to notable inaccuracies or ambiguities. Ultimately, this cross-sectional study included 3,506 participants for analysis (Figure 1).

This study was approved by the Research Ethics Committee, The First People’s Hospital of Baiyin. Participants were assured that any personal information obtained from the questionnaires would be kept confidential and used solely for research purposes. Informed consent was obtained from all participants before they completed the questionnaire.

### Questionnaire

The questionnaire for this study was meticulously developed following an exhaustive review of pertinent literature. It was structured to include both independent and dependent variables. The independent variables (IVs) were designed to capture a range of sociodemographic attributes of the student participants, including but not limited to age, gender, ethnicity, academic discipline, grade, the geographic location of their university (divided into Southwest China and Northwest China), the university’s Double 1st-Class status, home region, whether they are an only child, romantic relationships, and relationships with classmates. Additionally, the IVs included elements related to physical activity participation and exercise intensity maintained by the students. The dependent variable (DV) of the study was specifically focused on SA.

### Scales for questionnaire

#### Physical activity rating scale

The evaluation of EI in this study was facilitated by the Physical Activity Rating Scale (PARS-3), an instrument initially developed by Kimio Hashimoto, a distinguished psychologist at Kyushu University in Japan. The scale has since been adapted into a revised Chinese version by researcher Liang (23). The PARS-3 consists of three distinct components that assess exercise intensity, duration, and frequency of engagement over the preceding month. Each component is evaluated using a 5-point Likert scale, with slight variations in the scoring range to accommodate the nature of each item. Specifically, the exercise intensity and exercise frequency components are scored on a scale from 1 to 5, while the duration component uses a scoring system ranging from 0 to 4.

The aggregate score for physical activity was determined using the formula: total PA score = exercise intensity × duration time × exercise frequency. This method yields a cumulative score ranging from 0 to 100 points (17, 24, 25). Higher scores indicate a more robust engagement in physical activity (17, 25). Additionally, EI was categorized into three distinct levels according to the classification system established by Liang (20): low-intensity (≤ 19 points), moderate-intensity (20–42 points), and vigorous-intensity (≥ 43 points). In this study, the Cronbach’s *α* coefficient for the PARS-3 scale was 0.709, indicating the scale’s internal consistency.

#### Smartphone addiction scale-Chinese short version

All participants in the study completed the Smartphone Addiction Scale-Chinese Short Version (SAS-CSV) to gage the severity of compulsive internet use. Kwon et al. developed the original Smartphone Addiction Scale (SAS), which contains 33 items and 6 factors based on self-reporting (26). It was subsequently revised into the Smartphone Addiction Scale-Short Version (SAS-SV) (27), which is widely used to measure SA among college students (28, 29). The SAS-SV was created by screening out 10 items most closely associated with smartphone dependence from the SAS and using an expert rating method to form a single dimension. Chinese scholar Zhao translated the SAS-SV into Chinese and evaluated it to establish the SAS-CSV (29).

The scale employs a 6-point Likert scale system, ranging from 1 (strongly disagree) to 6 (strongly agree) points (1 = strongly disagree, 2 = disagree, 3 = somewhat disagree, 4 = somewhat agree, 5 = agree, 6 = strongly agree), with total scores ranging from 10 to 60 points. Higher scores indicate a greater tendency toward SA. The criteria for addiction are: if a female score ≥ 33, or a male score ≥ 31, they are classified as having a SA; otherwise, they are considered non-addicted (24). The Cronbach’s *α* coefficient for the scale is 0.911 (24). In the present sample, the scale exhibited excellent reliability, with a Cronbach’s α coefficient of 0.929, indicating a high level of internal consistency among the items.

#### Validity and reliability of scales

Two scales were used in this study: the Physical Activity Rating Scale (PARS-3) and the Smartphone Addiction Scale-Chinese Short Version (SAS-CSV). Both scales are widely used in research. The PARS-3 has been applied by many Chinese scholars in recent years (16, 30–32). In this study, the Cronbach’s *α* coefficient for the PARS-3 scale was 0.709, indicating the scale’s internal consistency. The SAS-SV has been widely used in both domestic and international research (33–35). Chinese scholar Zhao Hao (29) translated the SAS-SV into the SAS-CSV. In the present sample, the scale exhibited excellent reliability, with a Cronbach’s α coefficient of 0.929, indicating a high level of internal consistency among the items.

### Statistical analysis

The measurement data are reported as the median and interquartile range (IQR) for continuous variables, denoted as [M (P_25_, P_75_)], due to their non-normal distribution. The Mann–Whitney U test was utilized to evaluate relationships between two data groups. Categorical data were presented in terms of frequency (n) and percentage (%). The chi-square test or Fisher’s exact test, as appropriate, was employed to detect differences between two or more groups. A descriptive analysis was conducted on all participants.

Univariate and multivariate logistic regression analyses were undertaken to determine the odds ratios (ORs) with 95% confidence intervals (CIs) for exploring the relationships between PA, EI and SA. A total of three models were constructed. In Model 1, adjustments were made for potential confounding sociodemographic characteristics including age, sex, ethnicity, home region, and being an only child. Model 2 included additional adjustments for university-related variables such as university ranking, location, discipline, and grade. Model 3 was fully adjusted and further included social relations, consisting of romantic relationships and relationships with classmates. All statistical analyses were performed using the statistical software package R 4.2.1 (The R Foundation, Vienna, Austria)^2^ and the Free Statistics software (version 2.0: Beijing Free Clinical Medical Technology Co. Ltd., Beijing, China), which utilizes R as the underlying statistical engine and has a graphical user interface (GUI) developed in Python. A *p*-value of <0.05 (two-sided) was considered statistically significant.

## Results

### Baseline characteristics of participants

A total of 3,506 participants, aged 19.0 (19.0, 20.0) years, were included in the analysis. Of these, 1,905 (54.3%) participants had SA disorder. Tables 1, 2 illustrate the baseline characteristics of all participants, categorized by their PA and EI. Compared to the group that did not engage in PA, the group participating in PA had a higher proportion of male participants (*p* < 0.001), a higher percentage of STEM discipline students (*p* < 0.001), a higher proportion of freshmen (*p* < 0.001), a higher percentage of students not in a romantic relationship (*p* < 0.001), a higher proportion who got along well with classmates (*p* < 0.001), and a lower percentage of SA disorder (*p* < 0.001). However, there were no significant differences between the two or three groups in terms of ethnicity, double first-class university status, university location, home region, or only-child status. Similar results were observed when comparing the group with lower EI, with college students who had higher EI being more likely to be male, in STEM disciplines, first-year students, not in a romantic relationship, having great relationships with classmates, and less likely to have SA disorder.

**Table 1:** Baseline characteristics of participants (*n* = 3,506).

Variables		Total (*n* = 3,506)	Participated in PA		Mann Whitney test /chi square test	
			No (*n* = 940)	Yes (*n* = 2,566)	*Z*/*χ*^2^ value	*p* value
Age, M (P_25_, P_75_)		19.0 (19.0, 20.0)	20.0 (19.0, 20.0)	19.0 (19.0, 20.0)	−2.535	0.011
Sex, n (%)	Male	1,743 (49.7)	310 (33.0)	1,433 (55.8)	143.901	< 0.001
	Female	1,763 (50.3)	630 (67.0)	1,133 (44.2)		
Ethnicity, n (%)	Han	3,080 (87.8)	835 (88.8)	2,245 (87.5)	1.156	0.282
	Minority	426 (12.2)	105 (11.2)	321 (12.5)		
Double 1^st^ -class, n (%)	No	3,349 (95.5)	894 (95.1)	2,455 (95.7)	0.519	0.471
	Yes	157 (4.5)	46 (4.9)	111 (4.3)		
University location, n (%)	Southwest	3,401 (97.0)	912 (97.0)	2,489 (97.0)	0.001	0.973
	Northwest	105 (3.0)	28 (3.0)	77 (3.0)		
Discipline, n (%)	Medicine	1,115 (31.8)	279 (29.7)	836 (32.6)	16.906	< 0.001
	STEM	1,294 (36.9)	317 (33.7)	977 (38.1)		
	Others	1,097 (31.3)	344 (36.6)	753 (29.3)		
Grade, n (%)	1^st^	1,721 (49.1)	380 (40.4)	1,341 (52.3)	46.193	< 0.001
	2^nd^	1,366 (39.0)	420 (44.7)	946 (36.9)		
	3^rd^	238 (6.8)	86 (9.1)	152 (5.9)		
	4^th^	144 (4.1)	48 (5.1)	96 (3.7)		
	5^th^	37 (1.1)	6 (0.6)	31 (1.2)		
Home Region, n (%)	City	1,034 (29.5)	284 (30.2)	750 (29.2)	0.751	0.687
	Town	680 (19.4)	174 (18.5)	506 (19.7)		
	Village	1,792 (51.1)	482 (51.3)	1,310 (51.1)		
Only child, n (%)	No	2,418 (69.0)	645 (68.6)	1,773 (69.1)	0.074	0.786
	Yes	1,088 (31.0)	295 (31.4)	793 (30.9)		
Romantic relationship, n (%)	No	2,536 (72.3)	729 (77.6)	1,807 (70.4)	17.488	< 0.001
	Yes	970 (27.7)	211 (22.4)	759 (29.6)		
Classmates’ relationship, n (%)	Poor	46 (1.3)	17 (1.8)	29 (1.1)	81.809	< 0.001
	Average	1,543 (44.0)	527 (56.1)	1,016 (39.6)		
	Great	1,917 (54.7)	396 (42.1)	1,521 (59.3)		
SA, n (%)	No	1,601 (45.7)	352 (37.4)	1,249 (48.7)	34.957	< 0.001
	Yes	1,905 (54.3)	588 (62.6)	1,317 (51.3)		

SA, smart addiction; PA, physical activity; EI, Exercise Intensity; STEM, Science, Technology, Engineering and Math.

**Table 2:** Baseline characteristics of participants (*n* = 3,506).

Variables		Total (*n* = 3,506)	EI level (scores)			Kruskal-Wallis test /chi square test	
			Low (≤19) (*n* = 2,330)	Moderate (20–42) (*n* = 543)	Vigorous (≥43) (*n* = 633)	*H* /*χ^2^* value	*p* value
Age, M (P_25_, P_75_)		19.0 (19.0, 20.0)	20.0 (19.0, 20.0)	19.0 (19.0, 20.0)	19.0 (19.0, 20.0)	0.500	0.779
Sex, n (%)	Male	1,743 (49.7)	915 (39.3)	348 (64.1)	480 (75.8)	319.227	< 0.001
	Female	1,763 (50.3)	1,415 (60.7)	195 (35.9)	153 (24.2)		
Ethnic, n (%)	Han	3,080 (87.8)	2,072 (88.9)	472 (86.9)	536 (84.7)	8.942	0.011
	Minority	426 (12.2)	258 (11.1)	71 (13.1)	97 (15.3)		
Double 1^st^ -class, n (%)	No	3,349 (95.5)	2,222 (95.4)	520 (95.8)	607 (95.9)	0.412	0.814
	Yes	157 (4.5)	108 (4.6)	23 (4.2)	26 (4.1)		
Univ location, n (%)	Southwest	3,401 (97.0)	2,261 (97.0)	532 (98.0)	608 (96.1)	3.750	0.153
	Northwest	105 (3.0)	69 (3.0)	11 (2.0)	25 (3.9)		
Discipline, n (%)	Medicine	1,115 (31.8)	803 (34.5)	170 (31.3)	142 (22.4)	40.978	< 0.001
	STEM	1,294 (36.9)	800 (34.3)	225 (41.4)	269 (42.5)		
	Others	1,097 (31.3)	727 (31.2)	148 (27.3)	222 (35.1)		
Grade, n (%)	1^st^	1,721 (49.1)	1,092 (46.9)	298 (54.9)	331 (52.3)	27.788	< 0.001
	2^nd^	1,366 (39.0)	955 (41.0)	185 (34.1)	226 (35.7)		
	3^rd^	238 (6.8)	166 (7.1)	25 (4.6)	47 (7.4)		
	4^th^	144 (4.1)	98 (4.2)	28 (5.2)	18 (2.8)		
	5^th^	37 (1.1)	19 (0.8)	7 (1.3)	11 (1.7)		
Home Region, n (%)	City	1,034 (29.5)	678 (29.1)	172 (31.7)	184 (29.1)	1.593	0.810
	Town	680 (19.4)	458 (19.7)	100 (18.4)	122 (19.3)		
	Village	1,792 (51.1)	1,194 (51.2)	271 (49.9)	327 (51.7)		
Only child, n (%)	No	2,418 (69.0)	1,611 (69.1)	375 (69.1)	432 (68.2)	0.189	0.910
	Yes	1,088 (31.0)	719 (30.9)	168 (30.9)	201 (31.8)		
Romantic relationship, n (%)	No	2,536 (72.3)	1,728 (74.2)	386 (71.1)	422 (66.7)	14.477	< 0.001
	Yes	970 (27.7)	602 (25.8)	157 (28.9)	211 (33.3)		
Classmates’ relationship, n (%)	Poor	46 (1.3)	31 (1.3)	5 (0.9)	10 (1.6)	69.777	< 0.001
	Average	1,543 (44.0)	1,138 (48.8)	200 (36.8)	205 (32.4)		
	Great	1,917 (54.7)	1,161 (49.8)	338 (62.2)	418 (66.0)		

PA, physical activity; EI, Exercise Intensity; STEM, Science, Technology, Engineering and Math; SA, smart addiction.

### Relationship between PA, EI and SA

In the univariate logistic regression analysis, PA, described as a binary variable, showed an inverse association with SA (*OR* = 0.63, 95% CI = 0.54–0.74, *p* < 0.001; Table 3). More importantly, this association remained statistically significant even after adjusting for potential confounders (Table 4, model 3), with an *OR* of 0.70 (95% CI = 0.59–0.82, *p* < 0.001).

**Table 3:** Association of covariates and smartphone addiction risk.

Variables		*OR* value	95% CI	*p* value
Participated in PA, n (%)	No	1(Ref)		
	Yes	0.63	0.54–0.74	<0.001
EI level, n (%)	Low	1(Ref)		
	Moderate	0.74	0.62–0.89	0.002
	Vigorous	0.75	0.63–0.89	0.001
Sex, n (%)	Male	1(Ref)		
	Female	1.22	1.07–1.40	0.003
Ethnic, n (%)	Han	1(Ref)		
	Minority	1.02	0.83–1.25	0.874
Double 1^st^ class, n (%)	No	1(Ref)		
	Yes	1.10	0.80–1.53	0.545
University location, n (%)	Southwest	1(Ref)		
	Northwest	1.00	0.68–1.47	0.992
Discipline, n (%)	Medicine	1(Ref)		
	STEM	0.88	0.75–1.03	0.113
	Others	1.21	1.02–1.43	0.028
Grade, n (%)	1^st^	1(Ref)		
	2^nd^	1.16	1.01–1.34	0.042
	3^rd^	1.50	1.13–1.98	0.004
	4^th^	1.50	1.06–2.13	0.023
	5^th^	0.79	0.41–1.51	0.475
Home Region, n (%)	City	1(Ref)		
	Town	1.26	1.04–1.53	0.020
	Village	1.35	1.16–1.57	<0.001
Only child, n (%)	No	1(Ref)		
	Yes	0.82	0.71–0.95	0.008
Romantic relationship, n (%)	No	1(Ref)		
	Yes	0.92	0.80–1.07	0.287
Classmates’ relationship, n (%)	Poor	1(Ref)		
	Average	1.48	0.82–2.66	0.190
	Great	1.01	0.56–1.80	0.986
Age, (years)		1.07	1.01–1.12	0.017

PA, physical activity; EI, Exercise Intensity; STEM, Science, Technology, Engineering and Math; OR, Odds Ratio, CI, Confidence Interval.

**Table 4:** Associations among participation of PA, EI and SA in the multiple regression model.

Variable	Total	SA, n (%)	Crude		Adjusted (Model 1)		Adjusted (Model 2)		Adjusted (Model 3)	
			OR (95% CI)	*p* value	OR (95% CI)	*p* value	OR (95% CI)	*p* value	OR (95% CI)	*p* value
PA participation										
No	940	588 (62.6)	1(Ref)		1(Ref)		1(Ref)		1(Ref)	
Yes	2,566	1,317 (51.3)	0.63 (0.54–0.74)	<0.001	0.65 (0.56–0.76)	<0.001	0.66 (0.56–0.77)	<0.001	0.70 (0.59–0.82)	<0.001
EI level										
Low	2,330	1,323 (56.8)	1(Ref)		1(Ref)		1(Ref)		1(Ref)	
Moderate	543	268 (49.4)	0.74 (0.62–0.89)	0.002	0.78 (0.64–0.94)	0.009	0.78 (0.64–0.94)	0.010	0.81 (0.67–0.98)	0.034
Vigorous	633	314 (49.6)	0.75 (0.63–0.89)	0.001	0.79 (0.66–0.95)	0.014	0.78 (0.64–0.93)	0.008	0.83 (0.68–1.00)	0.046
*P* for trend	3,506			<0.001		0.004		0.002		0.020

Model 1: adjusted for sociodemographic variables (age, sex, ethnic, home region, only child). Model 2: adjusted for model 1 + Double 1^st^-class, University location, Discipline and Grade. Model 3: adjusted for model 2 + Romantic relationship and Classmates relationship. PA, physical activity; EI, Exercise Intensity; OR, Odd Ratio; 95% CI, 95% confidence interval.

Meanwhile, in the univariate logistic regression analyses, EI, expressed as a categorical variable with three levels, also demonstrated a negative association with SA. Specifically, compared with individuals with low EI, the crude ORs for moderate EI and vigorous EI were 0.74 (95% CI = 0.62–0.89, *p* = 0.002) and 0.75 (95% CI = 0.63–0.89, *p* = 0.001), respectively (Table 3). Furthermore, this association remained statistically significant, with *OR*s of 0.81 (95% CI = 0.67–0.98, *p* = 0.034) and 0.83 (95% CI = 0.68–1.00, *p* = 0.046), respectively, independent of potential confounders (Table 4, model 3). Moreover, in this context, with the increased in EI levels, there was a discernible trend toward a decrease in SA, *P* for trend was 0.020 (Table 4, model 3).

### Subgroup analysis

**Figure 2:**
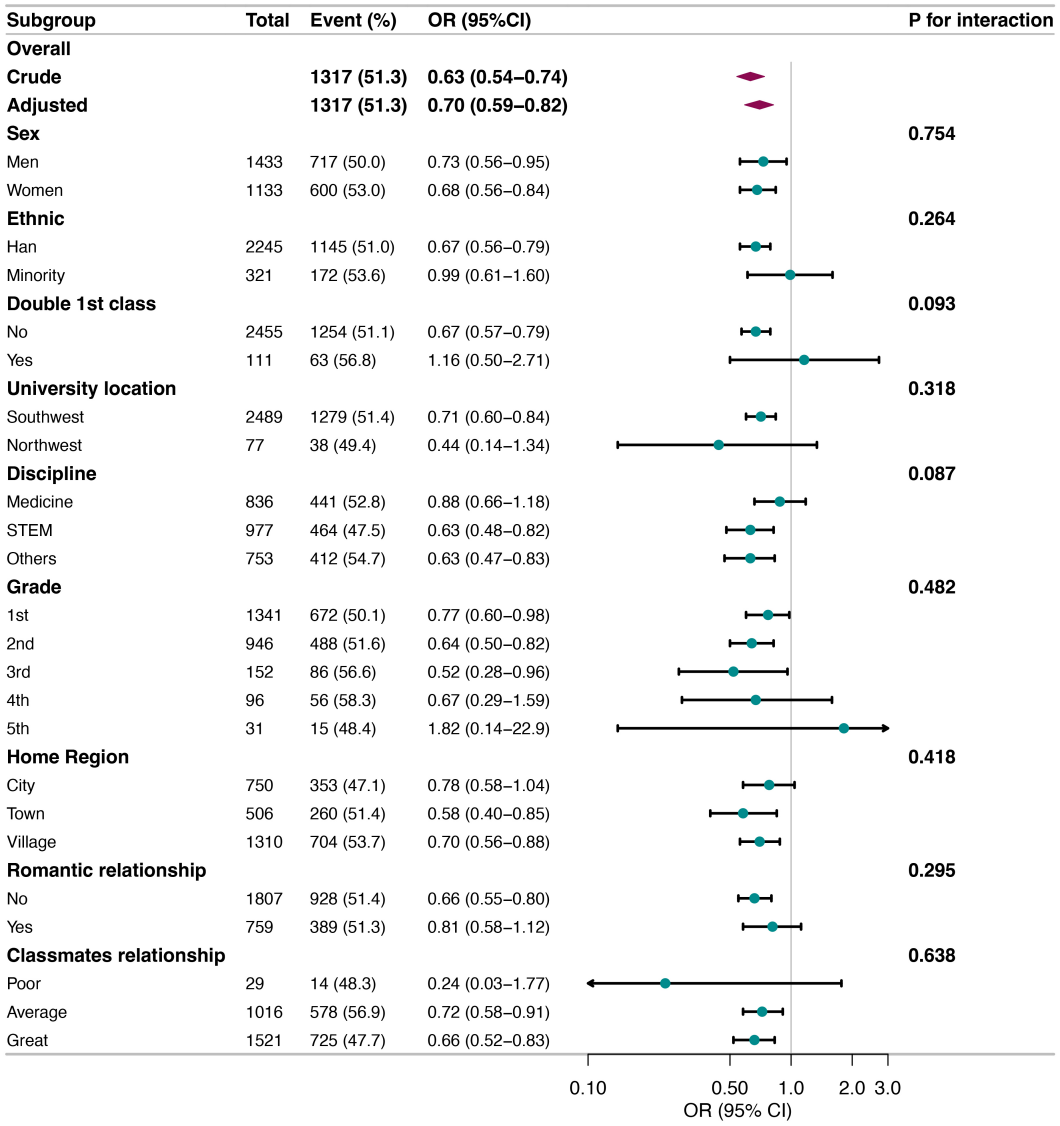
Forest plot. Association between PA and SA according to different subgroups. Each stratification factor was adjusted for all other variables, including age, sex, ethnicity, home region, only-child status, double first-class status, university location, discipline, grade, relationship status, and interpersonal relationships, except for the stratification component itself. STEM, Science, Technology, Engineering, and Math; OR, Odds Ratio; 95% CI, 95% Confidence Interval.

Further, we conducted stratified and interaction analyses to ascertain potential effect modification on the relationship between PA and SA (Figure 2). Consistent results were observed when the analysis was stratified by sex, ethnicity, double first-class status, university location, discipline, grade, home region, romantic relationship status, and classmates’ relationships. In addition, as shown in Figure 2, PA was associated with SA among college students who were female (*OR* = 0.68, 95% CI = 0.56–0.84), of Han nationality (*OR* = 0.67, 95% CI = 0.56–0.79), from non-double first-class universities (*OR* = 0.67, 95% CI = 0.57–0.79), located in northwest China (*OR* = 0.44, 95% CI = 0.14–1.34), in STEM disciplines (*OR* = 0.63, 95% CI = 0.48–0.82), junior students (*OR* = 0.52, 95% CI = 0.28–0.96), from towns (*OR* = 0.58, 95% CI = 0.40–0.85), not in a romantic relationship (*OR* = 0.66, 95% CI = 0.55–0.80), and had good relationships with classmates (*OR* = 0.66, 95% CI = 0.52–0.83). However, no statistically significant interactions were found in any subgroup analyses for effect modification, suggesting that the risk estimates for developing SA were largely consistent across different subgroups.

## Discussion

This study explores the link between PA, EI, and SA among college students in Western China. Findings reveal an inverse relationship: students engaging in higher-intensity PA had a lower risk of SA. This remained significant after adjusting for confounders, with subgroup analyses confirming the robustness of the results across demographic and lifestyle factors. Promoting PA may effectively manage SA, aligning with research on PA’s protective role against behavioral addictions like internet addiction (20, 36). The large sample size enhances the reliability of these findings.

Although IA and SA share similarities, a key distinction is that smartphones are more portable and convenient to use, whereas computers often provide greater power for gaming with enhanced psychological rewards. Supporting this, Rozgonjuk et al. found that while risk factors generally had stronger associations with problematic internet use than with smartphone use, fear of missing out was more strongly linked to problematic smartphone use (37).

Our findings are in line with previous studies from other regions in China, such as Central (38) and Northern China (39), showing a similar pattern among college students. For instance, research in Central China demonstrated a significant reduction in SA after students engaged in basketball and Baduanjin for 12 weeks, with benefits sustained for an additional 2 months (40). This effect, observed in students from a different geographical context, highlights the potential of PA as a preventive approach to SA across various Chinese ethnics/races, consistent with the idea that the benefits of PA in mitigating SA also apply to college students in Western China.

Findings from Taiwan region contrast with ours, as a six-month longitudinal study reported no significant link between PA and SA (41). These differences may reflect cultural variations in exercise habits, smartphone use, attitudes toward PA, or age. Similar inconsistencies between mainland China and Taiwan region in health behaviors (36, 42) suggest that distinct forms of PA or fitness norms among mainland students may influence the PA-SA link. Further research should explore these cultural differences to validate our findings.

The analysis of EI highlights PA’s role in managing SA, as students engaging in moderate-to-vigorous PA had significantly lower odds of SA. Higher-intensity PA may offer protective benefits by fostering resilience and improving mood, reducing smartphone dependency (43, 44). Vigorous PA promotes endorphin release, which buffers against stress and anxiety linked to SA (20).

The inverse association between PA and SA remained consistent across subgroups, including gender, ethnicity, academic discipline, and social factors, suggesting PA’s protective effect is broadly applicable. This indicates that PA’s impact on SA likely stems from fundamental psychological or physiological mechanisms, rather than context-specific factors. The similar benefits across relationship statuses and academic disciplines support PA as a general preventive measure, due to its wide-ranging mental health benefits (45, 46). This uniformity suggests that PA interventions can be widely implemented without extensive customization, making them accessible to large student populations.

Our comparisons with studies from Central and Northern China, as well as Taiwan region, highlight the importance of cultural and environmental contexts in PA and SA research. Mainland Chinese students may prioritize fitness differently from students in Taiwan, influenced by unique societal expectations, which could shape how PA affects SA. Cultural factors significantly influence health behaviors, explaining the differing impact of PA across these populations (36, 42). Future research should explore these cultural influences to tailor intervention programs to regional behaviors and preferences.

Specific interventions could include tertiary institutions promoting greater student participation in physical activity through educational campaigns, providing suitable facilities, and organizing competitive sporting events (47).

It is important to note that China is divided into six major regions—North China, Northeast China, East China, South Central China, Southwest China, and Northwest China—by the National Bureau. This regional division helps better understand the nation’s characteristics, including its economy, climate, geography, governance, and administrative divisions (48).

Our current findings were collected solely from Western China, which has a disadvantaged economic status, partly due to its geographic features, such as high mountains and desert regions, as well as lower educational opportunities. Students from these areas may face less academic pressure due to fewer “tiger parents” in the region. While our data provides valuable insights into PA and SA, it may not be generalizable to students across all of China or the world. To address this limitation, we plan to conduct future longitudinal studies with larger cohorts from various regions of China—Eastern, Western, Southern, Northern, and Central—using a well-designed approach. Furthermore, we will minimize bias in cohort recruitment by accounting for factors such as ethnicity, sex, age, study years, academic discipline, parental education levels, and professions. This methodology is expected to provide more reliable and representative outcomes that can be generalized more effectively.

Since self-response bias is a key issue in psychometrics, Rosenman et al. developed a unique stochastic frontier estimation (SFE) method that substantially reduces, if not eliminates, this bias (49). We plan to adopt this system in our future study.

Although the current study’s cross-sectional design cannot establish causality between PA and SA, it reveals a significant association. The data cannot confirm whether PA directly reduces SA or if students with lower SA are more likely to engage in PA. Unmeasured factors, such as personality traits, lifestyle habits, or support systems, may influence both PA and SA. Previous research emphasizes the importance of longitudinal and experimental designs to clarify causality in behavioral addiction studies (27, 50). Future research using longitudinal and intervention-based approaches could better assess the causal relationship between PA and SA.

Our findings suggest mechanisms through which PA might reduce SA, including stress reduction, mood enhancement, and increased social interaction. PA has been shown to decrease stress, anxiety, and depressive symptoms, which could reduce students’ reliance on smartphones as coping tools (43, 51). Team-based PA also provides opportunities for socialization, offering an alternative to smartphone-based interactions and potentially reducing excessive smartphone use (44). These psychological benefits support PA’s inclusion in mental health and SA prevention programs, reinforcing its role in student well-being.

## Conclusion

In conclusion, promoting PA, especially at higher intensities, could help manage SA among college students. By integrating these findings into university wellness programs, institutions can address SA and encourage healthier lifestyles. Future research should explore cultural variations, causative mechanisms, and tailored interventions to maximize PA’s impact on reducing SA.

## Data Availability

The raw data supporting the conclusions of this article will be made available by the authors, without undue reservation.

## Footnotes

^1^ https://www.wjx.cn/

^2^ http://www.R-project.org
