# Supplementary material for: Exploring the relationship between physical activity and smartphone addiction among college students in Western China
Source: Front Public Health. 2025 Feb 21;13:1530947. doi: 10.3389/fpubh.2025.1530947 (PMC11885492; doi:10.3389/fpubh.2025.1530947)
Supplement: Supplementary file 1 [file Table_1.docx]

Supplementary table 1. University affiliations of college students participating in the cross-sectional study.

| Geographic location | Name of college |
| --- | --- |
| Southwest | Sichuan University |
|  | the University of Electronic Science and Technology of China |
|  | Southwest Jiaotong University |
|  | Chengdu University of Traditional Chinese Medicine |
|  | Sichuan Agricultural University |
|  | Southwestern University of Finance and Economics |
|  | Chengdu University |
|  | Chengdu Medical College |
|  | Southwest Medical University |
|  | North Sichuan Medical College |
|  | Panzhihua College |
|  | China West Normal University |
|  | Neijiang Normal University, |
|  | Chengdu University of Technology |
|  | Southwest Petroleum University |
|  | Sichuan University of Science & Engineering |
|  | Chengdu Technological University |
|  | Sichuan Normal University |
|  | Chengdu University of Information Technology |
|  | Southwest University of Science and Technology |
|  | Geely University of China |
|  | Xichang University |
|  | Chengdu Normal University |
|  | Sichuan Vocational College of Finance and Economics |
|  | Southwest Minzu University |
|  | Leshan Normal University |
|  | Sichuan Minzu College |
|  | Chengdu Sport University |
|  | Chengdu Neusoft University |
|  | Yibin University |
|  | Chengdu College of Arts and Sciences |
|  | Chengdu Technological University |
|  | Mianyang Teachers’ College |
|  | Xihua University |
|  | Sichuan Conservatory of Music |
|  | Chengdu International Studies University |
|  | Chongqing University |
|  | Southwest University |
|  | Chongqing Institute of Foreign Studies |
|  | Chongqing Normal University |
|  | Sichuan International Studies University |
|  | Chongqing Technology and Business University |
|  | Chongqing Jiaotong University |
|  | Chongqing University of Posts and Telecommunications |
|  | Chongqing Three Gorges University |
|  | Chongqing University of Science & Technology |
|  | Chongqing University of Technology |
|  | Chongqing Medical University |
|  | Guizhou University |
|  | Zunyi Medical University |
|  | Southwest Forestry University |
|  | Kunming University of Technology |
|  | Yunnan University of Finance and Economics |
|  | Yunnan Arts University |
|  | Guangxi University |
|  | Guilin University of Technology |
|  | Guangxi Normal University |
|  | Guangxi Minzu University |
|  | Yulin Normal University |
|  | Guangxi Vocational & Technical College |
| Northwest | Lanzhou University |
|  | Lanzhou Jiaotong University |
|  | Lanzhou University of Technology |
|  | Lanzhou Institute of Technology |
|  | Tianshui Normal University |
|  | Xinjiang Medical University |
|  | Kashi University |
|  | Qinghai University |
|  | Qinghai Minzu University |
|  | Northwest A&F University |
|  | Shaanxi University of Technology |
|  | Xi’an University of Posts & Telecommunications |
|  | Chang’an University |
|  | Shaanxi Normal University |
|  | Shaanxi University of Science & Technology |
|  | Xi’an Jiaotong University |
|  | Xi’an University of Technology |
|  | Xi’an Medical University |
|  | Ankang University |
|  | Northwest University |
|  | Xi’an University of Finance and Economics |
|  | Xi'an Technology and Business College |
|  | Xidian University |
|  | Northwestern Polytechnical University |
|  | Tibet University |
